# Demographic differences in access to health/therapeutic services over first year of the pandemic: a SPARK COVID-19 impact survey analysis

**DOI:** 10.3389/frhs.2024.1343636

**Published:** 2024-04-30

**Authors:** J.-M. Tsai, A. N. Bhat

**Affiliations:** ^1^ Department of Physical Therapy, University of Delaware, Newark, DE, United States; ^2^ Interdisciplinary Neuroscience Graduate Program, University of Delaware, Newark, DE, United States; ^3^ Biomechanics & Movement Science Program, University of Delaware, Newark, DE, United States; ^4^ Department of Psychological & Brain Sciences, University of Delaware, Newark, DE, United States

**Keywords:** autism spectrum disorder (ASD), COVID-19 pandemic, speech language therapy (SLT), physical/occupational therapy (PT/OT), applied behavior analysis (ABA), mental health (MH) services, medical (MED) services

## Abstract

**Introduction:**

This analysis examined changes in services received and service recovery one-year post-pandemic compared to pre-pandemic levels in children with ASD aged between 19 months and 17 years in various subgroups based on factors such as age, income, race/ethnicity, geographic location, and sex.

**Methods:**

An online, parent report survey was completed by the parents of children with ASD in the SPARK study cohort (*N* = 6,393). Descriptive statistics, chi-square analyses, and Spearman correlations were performed to study associations between various factors and service access, pre-pandemic and one-year, post-pandemic.

**Results:**

One year after pandemic, the lag in service recovery in children with ASD was greatest for PT/OT services followed by SLT. ABA services only recovered in half of the subgroups. In contrast, SES fully recovered and MH and MED services superseded pre-pandemic levels. Across majority of the timepoints, younger children received more SLT, PT/OT, and ABA services whereas older children received more SES, MH, and MED services. Higher income families accessed more SES, SLT, and ABA whereas lower income families received more MH services. White families received less SLT compared to non-white families. Hispanic families received more SLT services compared to non-Hispanic families. Compared to rural families, urban families received more ABA services at baseline which also recovered one year after the pandemic. Certain counterintuitive findings may be attributed to home/remote schooling leading to reduced access to related services.

**Conclusions:**

Future research and policy changes are needed to address the American healthcare vulnerabilities when serving children with ASD by enhancing the diversity of healthcare formats for continued service access during future pandemics and other similar crises.

## 1 Introduction

Autism Spectrum Disorder (ASD) affects 1 in every 36 children in the United States (1). Due to various core (social communication and restricted/repetitive behaviors) and co-occurring (sensorimotor, language, and cognitive) impairments of ASD (2–5) families and children often access a variety of health services from early childhood to adulthood (6–8). Children with ASD commonly receive special education (SES), occupational therapy (OT), physical therapy (PT), speech language therapy (SLT), applied behavioral analysis (ABA), mental health (MH), and medical (MED) services including prescription medications (6, 9, 10). Pre-pandemic, service receipt rates reported in the literature for children with ASD varied across studies with 85%–97% individuals receiving SES, 35%–80% receiving OT, 32%–35% receiving PT, 45%–71% receiving SLT, 21%–40% receiving ABA, 42%–46% receiving MH, and 48%–56% receiving MED services (6, 7, 9, 11–14). Although 74% of children with ASD benefit from a wide range of services in the school settings, there were still ∼63% children seeking services outside of school (7, 15). SLT and PT/OT were most accessed services in school settings, whereas ABA, MH, and MED services were often accessed outside of school (7, 15, 16).

Various demographic factors, such as child age and family's socioeconomic status (SES), race, ethnicity, and geographic location (urban/rural) are associated with access to services in children with ASD and related disorders (7, 11–13). For instance, access to some services (e.g., SLT, PT/OT, and ABA) reduces with age (13); higher socioeconomic status (i.e., higher family income) is associated with greater use of SLT, ABA, MED, SES and MH services (15–20); Hispanic children are less likely to receive SES, SLT, OT, and MED services compared to non-Hispanic children (17, 21–25). Last but not the least, children living in metropolitan areas (vs. rural areas) were found to have greater access to SLT and behavioral therapies (7, 16). These demographic inequities in service access were further tested during the recent COVID-19 pandemic.

The COVID-19 pandemic was marked by sudden school and facility closings due to strict lockdown throughout the US. Most healthcare services were negatively impacted, rendering significant and long-lasting disruptions of available services for children with developmental disabilities and their families (26–29). Jeste and colleagues reported 63%–70% of families lost various therapeutic services (26). Children with ASD had even more significant service disruptions. The study team at the Simons Foundation Powering Autism Research for Knowledge (SPARK) conducted online surveys in a large sample of parents and caregivers of children with ASD between April 2020 and July 2021. 78.5% families reported moderate to severe disruptions due to the pandemic (27, 28) 80% families reported disruptions to SES, 88% to SLT, 84% to PT/OT, and 77% to ABA services (27, 28). Younger children and children from low-income families faced greater service disruptions and significant parental stress due to pandemic-related loss in services (27, 28–31). Currently, there are limited data on how services recovered over the first year following the pandemic. Hence, we describe data from the SPARK COVID impact survey on how services recovered at the end of the first year of the pandemic (March 2021) compared to pre-pandemic service levels offered to children with ASD. In addition, we examine the variations in services received based on multiple demographic factors, such as age, family income, race/minority status, ethnicity, geographic location, and sex. Based on past literature, we hypothesize that younger children, children from racial/ethnic minorities, low-income families, and rural areas, will show differences in service recovery one-year post-pandemic compared to other demographic groups.

## 2 Methods

### 2.1 Participants

At the onset of the COVID-19 pandemic in March 2020 (timepoint 1 or T1) parents of children with ASD from the SPARK study completed the COVID-19 impact survey. These families were re-contacted at multiple additional timepoints in April 2020 (time point 2 or T2), August 2020 (time point 3 or T3), October 2020 (time point 4 or T4), March 2021 (time point 5 or T5), and July 2021(time point 6 or T6) to obtain data on their access to various health/therapeutic services (see Table 1). In this report, we focus on data related to service receipt in children and adolescents between 19 months and <18 years of age. For a multiplex family (i.e., family with more than one child with ASD), a single child was selected at random by the parent and their issues have been reported for all timepoints. Note that T1 data on service disruptions at the onset of the pandemic have already been analyzed and reported by White et al. (27) and Bhat (28).

**Table 1:** SPARK study sample size over time based on inclusion/exclusion criteria.

Time of survey administration	March 2020 (T1)	April 2020 (T2)	August 2020 (T3)	November 2020 (T4)	March 2021 (T5)	July 2021 (T6)
Question about services received/access	Not asked; Disruptions reported in Bhat (28)	Pre-pandemic (Jan/Feb 2020)	During last 2 months	During last 2 months	During last 2 months	During last 2 months
Original dataset	9,249	4,461	3,620	3,274	2,885	2,501
Form completed	9,027	4,370	3,620	3,274	2,885	2,501
Age ≤18 years	7,889	3,742	3,059	2,786	2,428	2,052
SCQ score non-blank	7,796	3,697	3,020	2,749	2,394	2,025
SCQ score ≥ 12	6,393	3,184	2,617	2,391	2,082	1,756
Final dataset	6,393	3,184	2,617	2,391	2,082	1,756
Total excluded	2,856 (30.9%)	1,277 (28.6%)	1,003 (27.7%)	883 (27.0%)	803 (27.8%)	745 (29.8%)

### 2.2 SPARK original procedures and data access

Families in the US with one or more children with ASD were eligible to join the SPARK study, an ongoing nationwide study following the development of individuals with ASD. Participants had been recruited through a growing number of clinical sites across the country using a multi-pronged social media strategy (32). Families voluntarily signed up for this study to fill out multiple online questionnaires on the SPARK website. This research team signed up for the SPARK study to gained access to the COVID-19 impact survey data and other demographic, medical, and clinical/developmental surveys completed by the participating families. This secondary data analysis was part of an exempt protocol approved by the University of Delaware Human Subjects Review Board (Protocol #: 1794596).

### 2.3 SPARK forms and measures

The SPARK COVID-19 impact survey asked families to report services received by their children and adolescents with ASD over the course of the pandemic including special education (SE), speech and language therapy (SLT), physical and occupational therapy (PT/OT), Applied Behavioral Analysis (ABA), Mental Health Services (MH), and medical services (MED). Note that the first data point from March 2020 (T1) only asked about service disruptions following the pandemic and these data have been reported in reference 28; hence, not discussed here.

In April 2020 or T2, parents were asked to recall the following, “Thinking back to January 2020, was your child regularly receiving the following service: ______”, for example, SES or SLT, and so on (Response: Yes = 1, No = 0, Missing Data = 888); hence, timepoint 2 or T2 provided data on pre-pandemic service levels. At 4 timepoints, August 2020 (T3), November 2020 (T4), March 2021 (T5) and July 2021 (T6), the following question was asked, “In the past 2 months, has the subject received the following service: _______”, for example, SES, SLT, and so on (Response: Yes = 1, No = 0).

The SPARK team also asked participating families to complete the basic medical screening form, the individual data form, and the Social Communication Questionnaire (SCQ). The basic medical screening form includes demographic information, birth history, professional diagnosis of ASD and other disorders, as well as other general medical conditions. The individual data form provides details on whether there is a presence of a cognitive impairment whether there is an Individualized Education Plan (IEP) for the child, and whether the child receives ASD services. Lastly, the Social Communication Questionnaire—Lifetime (SCQ) is a widely used parent questionnaire (Yes/No format) to screen for autistic traits in children above 4 years of age with a mental age of at least 2 years (33). A total SCQ score of >12 is indicative of a social communication delay and the child has a greater likelihood of being on the autism spectrum. The 12-point cut-off is a research recommended, more sensitive cut-off score (34–37). However, the timepoint T6, reflected service levels after summer school closings in July 2021; for this reason, we will focus on comparing timepoints T2 (pre-pandemic) and T5 (March 2021, 1 year after the pandemic). T5 was the latest timepoint during the school year when regular services were offered.

**Percent services received** at any given timepoint was the proportion of families receiving a particular service—SES, SLT, PT/OT, ABA, MH, or MED.

#### 2.3.1 Service recovery

We defined full service recovery at any given timepoint, if the service was ≥85% of the percent service receipt at baseline/pre-pandemic. Note that small differences are not being considered as true because this is a cross-sectional analysis and services may differ due to differences unique to the respondents at each timepoint. Percent service recovery was calculated as the percentage of services received by a given subgroup at timepoint 5 (T5%) divided by the percentage of services received, pre-pandemic (T2%), multiplied by 100.

### 2.4 Inclusion/exclusion criteria

Table 1 lists the exclusion criteria in the first column as well as the basis for data exclusion/filtering. Out of the originally contacted families (*N* = 9,249), we only included those who completed the COVID-19 survey and who reported their child being younger than 18 years; which results in a sample of 7,889. We limited the sample to under 18 years in order to assess children who have access to IDEA-based services such as PT, OT, SLT; which is often provided to school-age children and not adults. Furthermore, only participants who completed the SCQ and met the SCQ cut-off of ≥12; often used to screen for ASD in large-scale studies (34, 35); which resulted in a final sample of 6,393.

Demographics of the final sample are presented in Table 2. Key demographics for this sample are as follows: ∼81% are males, ∼69% are Caucasian, ∼18% are multi-racial, 16% are Hispanics, there is a fairly equal distribution of income from ≤20K to ≥161K USD (6%–12%), ∼67% are from urban areas, and there is a fairly equal distribution of age from 3 to 18 years (i.e., 15%–24%) with ∼1.4% belonging to <3 years of age. Most of the missing data was for information on geographic location (∼21%), income (∼11%), and race (∼4%).

**Table 2:** SPARK study demographic information for the sample.

N	6,393
Sex	Female: 1,235 (19.3%), Male: 5,158 (80.7%)
Minority status	White (includes families with at least one white parent): 5,464 (85.5%), Non-white: 659 (10.3%), Missing: 270 (4.2%)
Race	More than one: 1,182 (18.5%), Asian: 104 (1.6%), African American: 262 (4.1%), Native American: 23 (0.36%), Native Hawaiian: 5 (0.08%), Other: 120 (1.9%), White: 4,427 (69.2%), Missing: 270 (4.2%)
Ethnicity	Not Hispanic: 5,350 (83.7%) Hispanic: 1,043 (16.3%)
Annual household income [$]	≤20K: 627 (9.8%), 21K–35K: 782 (12.2%), 36K–50K: 729 (11.4%), 51K–65K: 608 (9.5%), 66K–80K: 667 (10.4%), 81K–100K: 667 (10.4%), 101K–130K: 657 (10.3%), 131K–160K: 362 (5.7%), ≥161K: 576 (9.0%), Missing: 718 (11.2%)
Geographic location	*Urban* [Large Central Metro: 1,121 (17.5%), Large Fringe Metro: 1,543 (24.1%), Medium Metro: 1,079 (16.9%), Small Metro: 543 (8.5%)], *Rural* [Micropolitan: 462 (7.2%), Noncore: 294 (4.6%)], Missing: 1,351 (21.1%)
Age A [Years]	A ≤ 3: 89 (1.4%), 3 < A ≤ 6: 1,136 (17.8%), 6 < A ≤ 9: 1,548 (24.2%), 9 < A ≤ 12: 1,447 (22.6%), 12 < A ≤ 15: 1,236 (19.3%), 15 < A ≤ 18: 937 (14.7%)

### 2.5 Statistical analysis

Statistical analyses were conducted using JMP Pro 16.0 (JMP, Inc). Proportion of different services received were compared across various demographic factors of sex, age, minority status, ethnicity, geographic location, socioeconomic status using chi-square analyses. Spearman correlation coefficients were used to examine associations between services received and various aforementioned factors. Statistical significance was set at *p* < 0.05. Bonferroni corrections were used when multiple comparisons/correlations were performed.

## 3 Results

### 3.1 Overall services received

**Figure 1:**
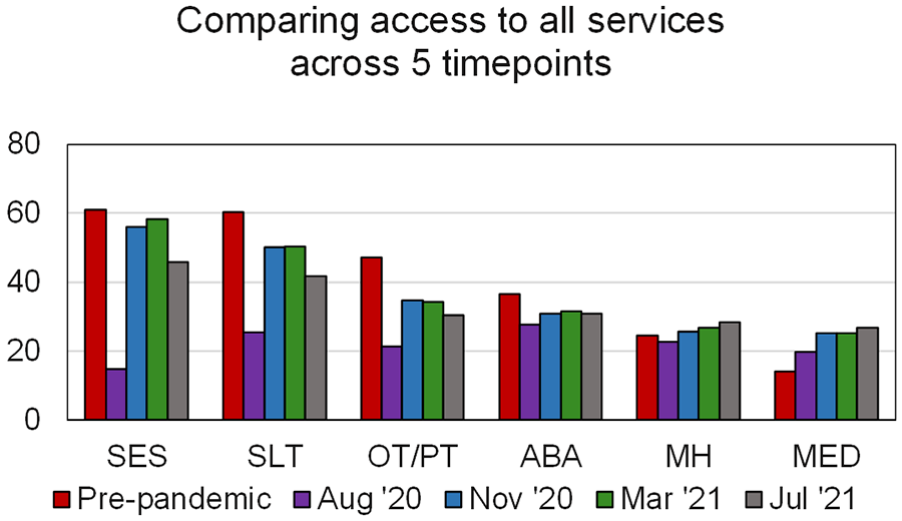
Percent of services received at various timepoints, pre-pandemic to July 2021. The Y-axis indicates % services received; the X-axis shows the various service types.

As shown in Figure 1, pre-pandemic (T2), 61.1% children and adolescents received SES, 60.2% received SLT, 47.1% received PT/OT, 36.6% received ABA, 24.6% received MH and 14.0% received MED services. At the onset of the pandemic in March 2020, most services were significantly reduced or came to a halt. Even by August 2020 (T3), based on trends shown in Figure 1 compared to pre-pandemic levels, school-based services such as SES, SLT, PT/OT, and ABA were substantially lower in receipt, and recovered gradually by March 2021 (T5). Note that services reduced slightly more in July 2021 (T6) due to summer time school closures.

### 3.2 Variations in services received based on age

**Figure 2:**
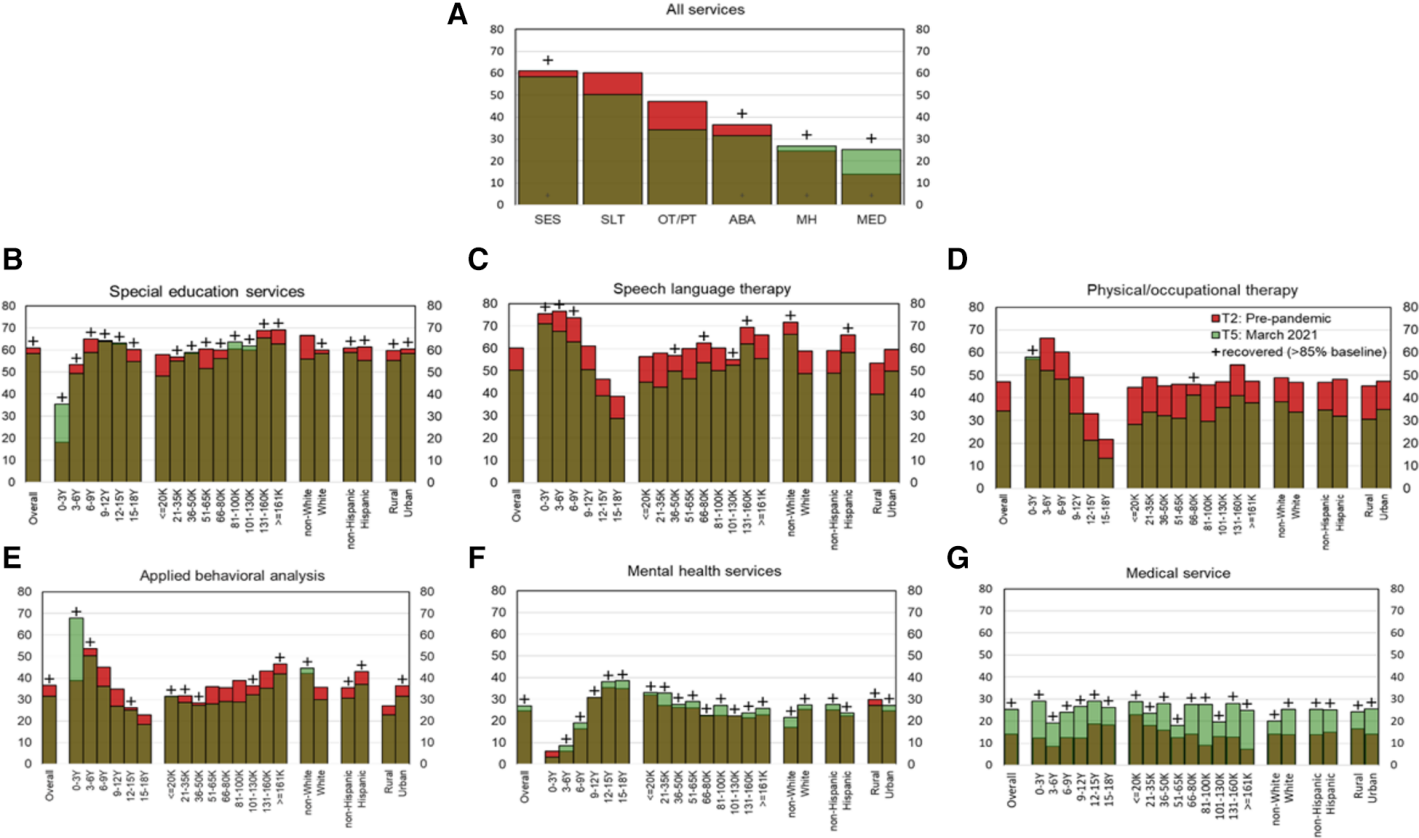
Service Recovery for the entire sample and for subgroups based on various demographic factors, is shown in (**A**) for all services, (**B**) for SES, (**C**) for SLT, (**D**) for PT/OT, (**E**) for ABA, (**F**) for MH, and (**G**) for MED services. Red bars indicate lag in service recovery and green bars indicate services surpassing pre-pandemic levels. The Y-axes in all panels shows percent service received. The X-axis in (**A**) indicates various service types and the X-axes in (**B-G**) indicate subgroups based on various demographic factors.

**Figure 3:**
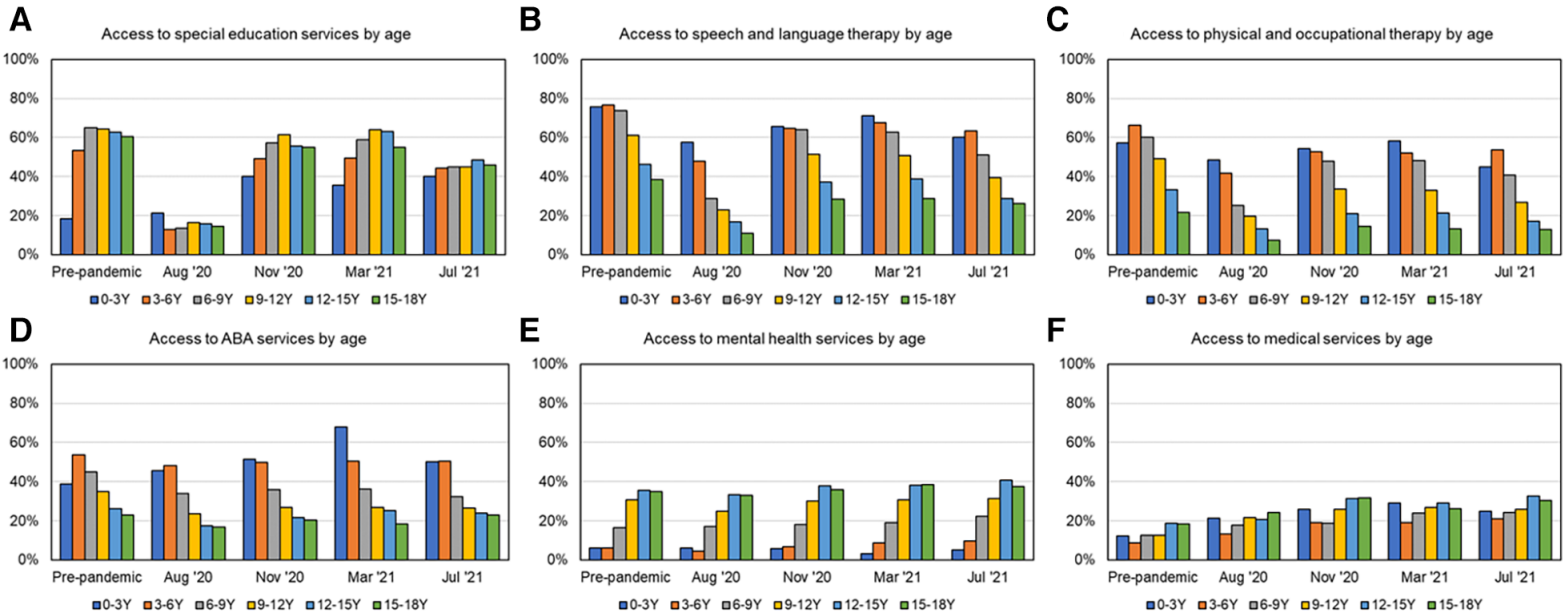
Percent services received as a function of age across timepoints over the first year of the pandemic, as shown in (**A**) for SES, (**B**) for SLT, (**C**) for PT/OT, (**D**) for ABA, (**E**) for MH, and (**F**) for MED services.

Figure 2 shows the comparisons of % service receipt between pre-pandemic baseline and 1 year-post-pandemic for each subgroup, based on age as well as various demographic factors. Table 3 shows the final % service recovery value for each subgroup as defined in section 2.3.1. As shown in Figure 2 and Table 3 for service recovery and Figure 3 on age-related differences in percent services received, for most time points, SLT, PT/OT, and ABA are provided more to younger children than older children with ASD as seen by the lowering trends with age. SES was relatively equal across school ages; however, access to MH and MED services increased with age. Post-pandemic, service recovery by March 2021 was seen in most age groups for SES, MH, and MED services but not for SLT, PT/OT, and ABA. Note that for ABA services, only three out of six age groups fully recovered (0–3Y, 3–6Y, 12–15Y). In fact, MED services showed more systematic scaling up in service proportions with age.

**Table 3:** Service recovery.

Services received/factors	Sub-group	SES	SLT	PT/OT	ABA	MH	MED
Age group	0–3 Years	193.2%	94.0%	101.6%	174.7%	52.7%	237.1%
	3–6 Years	92.6%	88.4%	78.4%	93.9%	145.9%	221.8%
	6–9 Years	90.6%	85.3%	80.0%	80.4%	115.7%	190.3%
	9–12 Years	99.3%	82.8%	67.1%	76.7%	100.3%	215.6%
	12–15 Years	100.6%	83.7%	64.5%	96.2%	107.4%	156.3%
	15–18 Years	91.0%	74.6%	60.7%	80.7%	110.4%	142.5%
Income level	<=$20K	83.1%	79.9%	63.4%	100.5%	103.3%	126.3%
	$21–35K	96.7%	73.8%	68.3%	90.5%	121.0%	130.5%
	$36–50K	100.9%	87.6%	71.0%	95.7%	105.5%	176.6%
	$51–65K	85.1%	77.4%	67.2%	77.4%	111.6%	143.0%
	$66–80K	93.4%	86.4%	89.6%	81.9%	100.9%	196.0%
	$81–100K	104.9%	83.2%	65.0%	74.3%	120.4%	303.8%
	$101–130K	103.0%	95.4%	76.2%	88.5%	99.5%	150.6%
	$131–160K	94.9%	89.0%	75.2%	81.4%	111.0%	218.6%
	>=$161K	90.5%	84.1%	79.6%	89.9%	113.3%	337.6%
Minority status	White	97.2%	82.8%	72.0%	83.8%	107.8%	183.0%
	Non-White	83.8%	92.3%	78.0%	106.2%	126.9%	143.6%
Ethnicity	Non-Hispanic	96.6%	82.8%	73.9%	86.2%	110.0%	182.5%
	Hispanic	90.1%	87.8%	66.2%	86.0%	105.8%	169.1%
Geographic location	Urban	96.5%	83.6%	73.4%	86.5%	110.1%	181.7%
	Rural	92.3%	73.9%	67.4%	84.3%	91.1%	147.3%
Sex	Male	94.3%	82.8%	71.1%	84.2%	109.4%	186.3%
	Female	100.5%	87.0%	78.8%	94.5%	108.7%	160.8%
Overall	Not applicable	95.6%	83.7%	72.7%	86.2%	109.4%	180.3%

SE, special education; SLT, speech and language therapy; PT/OT, physical and occupational therapy; ABA, applied behavior analysis; MH, mental health; MED, medical. Gray shading indicates the subgroup did not show service recovery one-year post-pandemic (T5) vs. pre-pandemic (T2). Percent service recovery was calculated as the percentage of services received by a given subgroup at one-year post-pandemic (T5) compared to the same at pre-pandemic baseline (T2) multiplied by 100.

### 3.3 Variations in services received based on income

**Figure 4:**
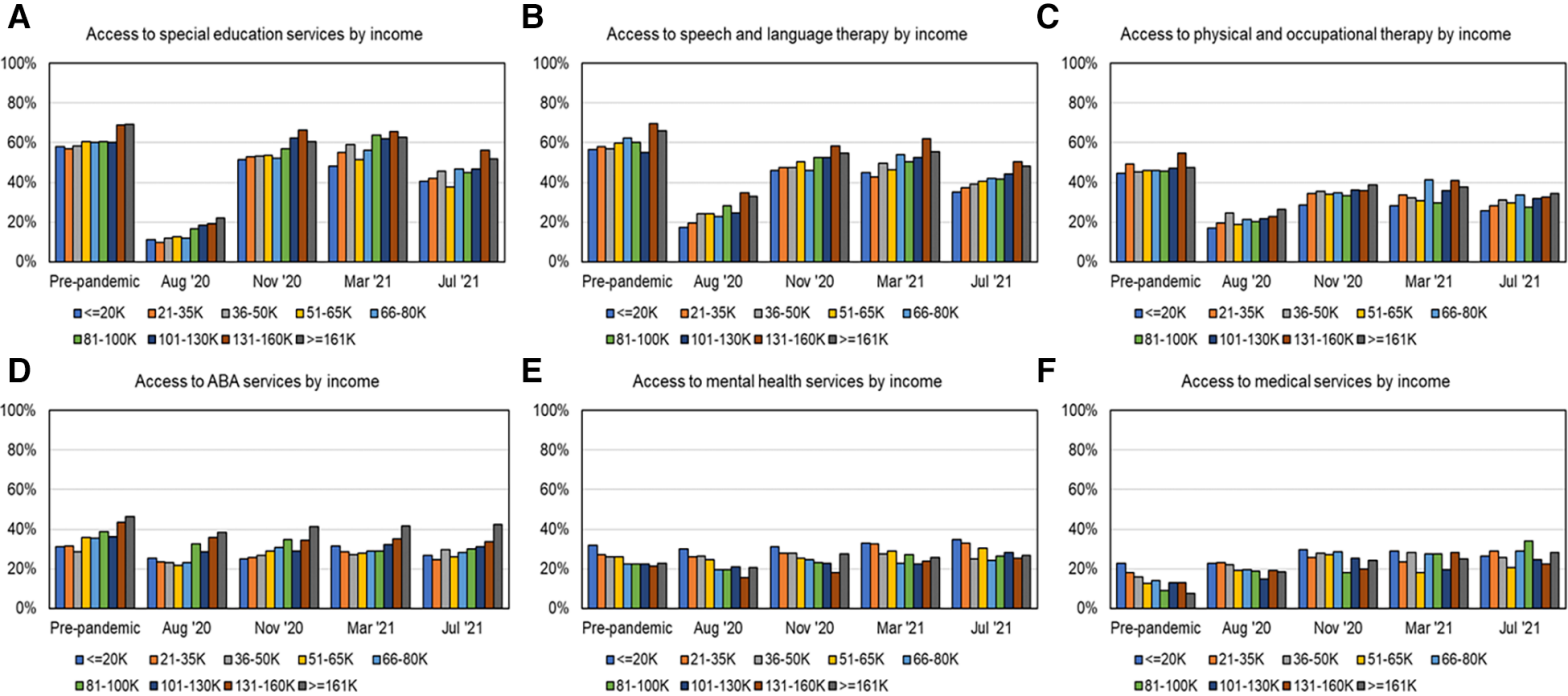
Percent services received as a function of income across timepoints over the first year of the pandemic, as shown in (**A**) for SES, (**B**) for SLT, (**C**) for PT/OT, (**D**) for ABA, (**E**) for MH, and (**F**) for MED services.

As shown in Figure 2 and Table 3 for service recovery and Figure 4 for income-based service proportions across multiple timepoints, SES, SLT, and ABA were accessed more by higher income families whereas lower income families accessed MH and MED services more. Post-pandemic, service recovery by March 2021 was seen in most income groups for SES, MH, and MED services but not for SLT, PT/OT, and ABA. As shown in Figure 2 and Tables 3, 5 out of 9 income groups did not fully recover in SLT services, 8 out of 9 income groups did not fully recover PT/OT services, and 4 out of 9 income groups did not fully recover in receiving ABA services. It must be noted that post-pandemic, access to MED services increased for all income groups.

### 3.4 Variations in services received based on minority status

**Figure 5:**
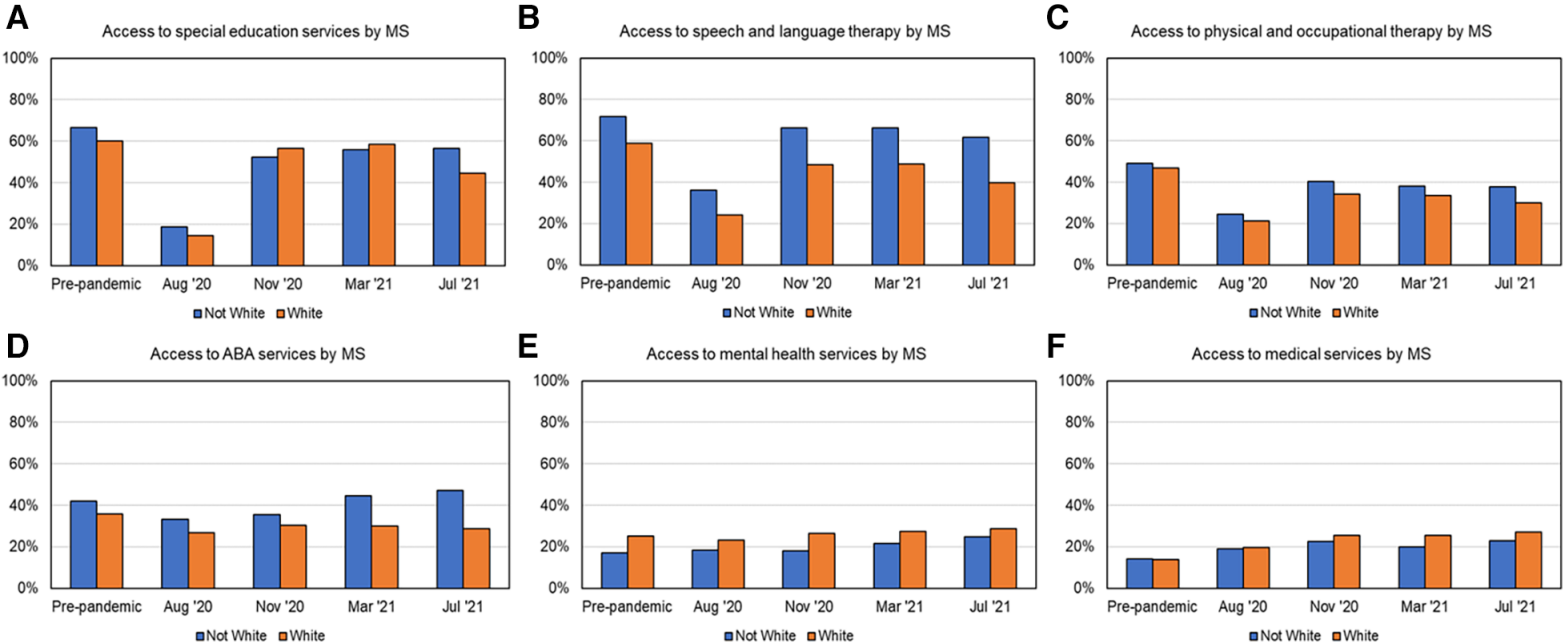
Percent services received as a function of minority status across timepoints over the first year of the pandemic, as shown in (**A**) for SES, (**B**) for SLT, (**C**) for PT/OT, (**D**) for ABA, (**E**) for MH, and (**F**) for MED services.

As shown in Figure 2 and Table 3 for service recovery and Figure 5 for services received by minority status across multiple timepoints, SLT, OT/PT and ABA services were accessed more by non-white families than white families. In contrast, MH and MED services were accessed more by white families than non-white families. As shown in Figure 2 and Table 3, post-pandemic service recovery by March 2021, was seen in both groups (non-white and white) for MH and MED services but did not occur for PT/OT services. SES recovered for white families whereas SLT and ABA services recovered for non-white families.

### 3.5 Variations in services received based on ethnicity

**Figure 6:**
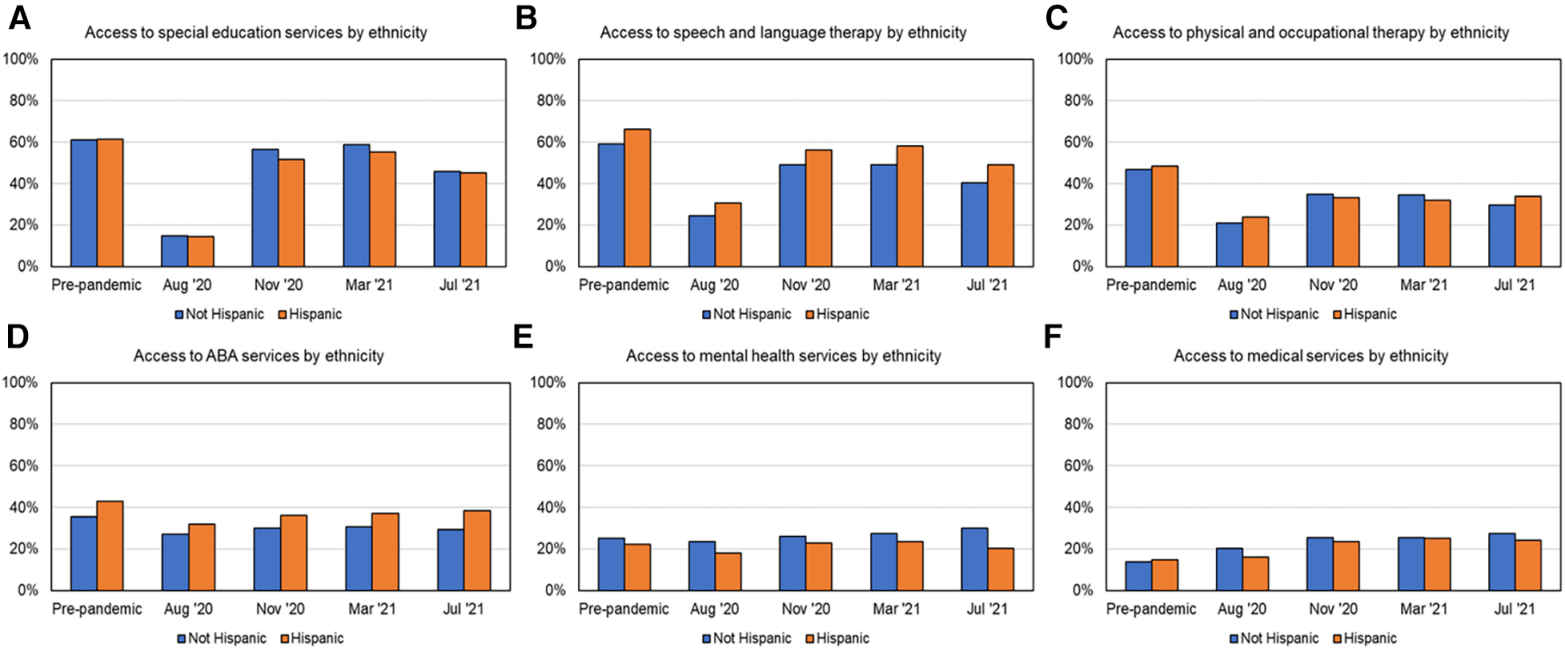
Access to services over time based on ethnicity, as shown in (**A**) for SES, (**B**) for SLT, (**C**) for PT/OT, (**D**) for ABA, (**E**) for MH, and (**F**) for MED services.

As shown in Figure 2 and Table 3 on service recovery and Figure 6 for services received based on ethnicity, across multiple timepoints, Hispanic families received more SLT and ABA services, compared to non-Hispanic families whereas non-Hispanic families received more MH services compared to Hispanic families. As shown in Figure 2 and Table 3, post-pandemic, service recovery for Hispanic families was seen for the majority of services except for PT/OT services whereas non-Hispanic families had full service recovery for most services except SLT and PT/OT services.

### 3.6 Variations based on geographic location and sex

**Figure 7:**
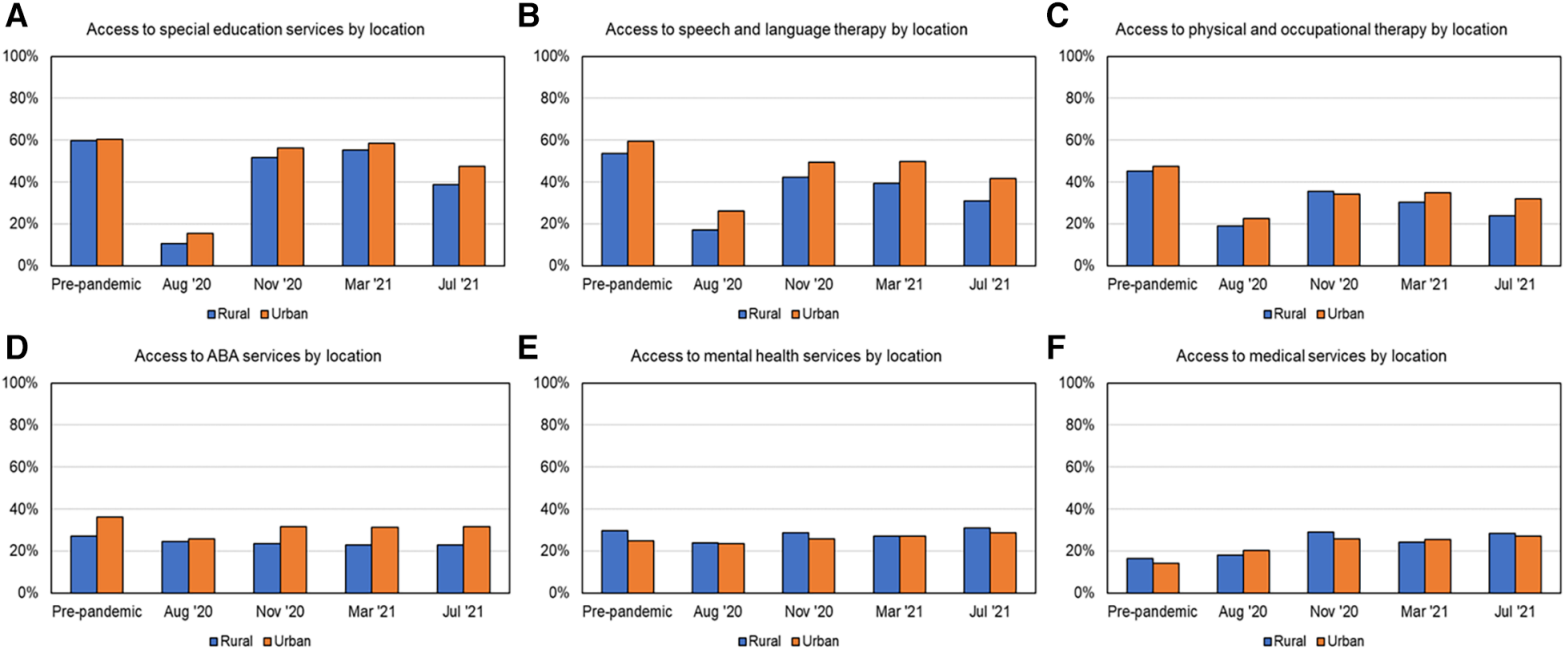
Access to services over time based on location, as shown in (**A**) for SES, (**B**) for SLT, (**C**) for PT/OT, (**D**) for ABA, (**E**) for MH, and (**F**) for MED services.

**Figure 8:**
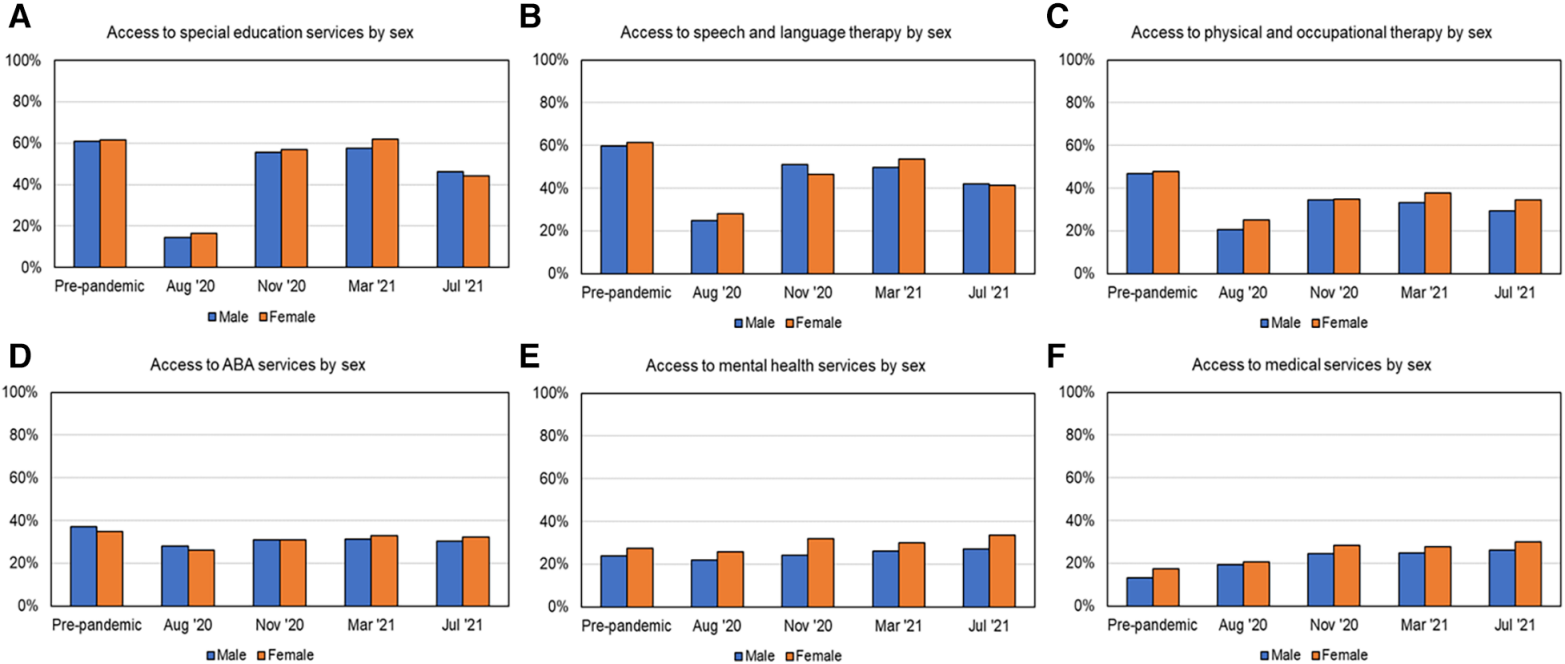
Access to services over time based on sex, as shown in (**A**) for SES, (**B**) for SLT, (**C**) for PT/OT, (**D**) for ABA, (**E**) for MH, and (**F**) for MED services.

As shown in Figures 2, 7, 8 and Table 3 at most time points, urban families received more SES, SLT, PT/OT, and ABA; whereas rural families received slightly more MH and MED services. As shown in Figure 2 and Table 3, post-pandemic service recovery was seen in majority of services for both urban and rural families; except SLT, PT/OT, and ABA services. SLT and PT/OT services did not recover for either geographic location groups. ABA recovered in urban but not the rural families. SES, MH, and MED services recovered in both groups based on location. Finally, there were sex-based differences in service recovery with males showing less recovery than females for SLT and ABA services and both groups not showing recovery for PT/OT services.

### 3.7 Chi-square analysis to examine service variations based on demographic factors

This analysis examined % service receipt at 1-year post-pandemic (T5%), compared to pre-pandemic baseline (T2%), and how that differed across demographic subgroups. Only T2 and T5 timepoints were compared as both timepoints occur during the school year. In addition, T2 was the earliest timepoint with service receipt data and T5 was the latest timepoint within the 1-year period. As shown in Figure 2 and Table 3 on service recovery and Table 4 on chi-square analyses indicated that access to multiple services differed across various demographic factors except sex. Majority of services including SES, SLT, PT/OT, ABA, MH services differed between age groups (pre-pandemic or timepoint 2 and 1-year post-pandemic or timepoint 5), whereas MED services only differed between age groups at T2 (pre-pandemic). Pre-pandemic, younger children with ASD received more SLT, PT/OT, and ABA services and relatively less SES, MH, and MED services compared to older children. One year after the pandemic, these trends did not change except for access to MED services did not differ between age groups. Based on income levels, pre-pandemic, higher income families accessed more ABA and less MED services compared to lower income families. However, these differences were not seen one year after the pandemic. Based on minority status, pre-pandemic, non-white families accessed more SLT services but fewer MH services compared to white families. One year after pandemic, non-white families continued to access more SLT and received more ABA services compared to white families. Based on ethnicity, one year after the pandemic, Hispanic families accessed more SLT services compared to non-Hispanic families. Based on geographic location, pre-pandemic, urban families accessed more ABA services but less MH services compared to rural families. One-year post-pandemic, urban families continued to access more SLT and ABA services compared to rural families. The non-significant results indicate that there was no significant difference in % service receipt between subgroups based on any given demographic factors. For exmaple, there were no sex-based differences in services received at either time points.

**Table 4:** Chi-squared values for services received (columns 3–8) in groups based on 6 demographic variables (rows 2–7) pre-pandemic (T2, first entry in each data cell) and march 2021 (T5, 12 months after pandemic onset—second entry in each data cell). Chi-squared values for *p* values less than adjusted alpha values are highlighted in yellow.

Services received/factors	Time point	SES	SLT	PT/OT	ABA	MH	MED
Age group	T2	119	220.4	269.1	119.2	316.6	52.03
	T5	29.46	166.9	205.9	116.5	149.7	NS
Income level	T2	NS	NS	NS	28.45	NS	53.24
	T5	NS	NS	NS	NS	NS	NS
Minority status	T2	NS	11.59	NS	NS	15.84	NS
	T5	NS	22.84	NS	17.38	NS	NS
Ethnicity	T2	NS	NS	NS	NS	NS	NS
	T5	NS	9.048	NS	NS	NS	NS
Geographic location	T2	NS	NS	NS	10.43	6.3	NS
	T5	NS	7.73	NS	6.6	NS	NS
Sex	T2	NS	NS	NS	NS	NS	NS
	T5	NS	NS	NS	NS	NS	NS

NS, not significant; SE, special education; PT/OT, physical and occupational therapy; SLT, speech and language therapy; ABA, applied behavior analysis; MH, mental health; MED, medical.

### 3.8 Correlations between various demographic factors and services received

Spearman correlations indicated that child's age, family income, minority status, ethnicity, and geographic location were significantly associated with % services received, as shown in Table 5 and Figures 3–8, albeit the correlation coefficients were relatively small. Pre-pandemic, child's age negatively correlated with access to SLT, PT/OT, and ABA services (i.e., younger children received more of these services), but positively correlated with access to SES, MH and MED (i.e., older children received more of these services). Similar trends were observed one year after the pandemic except for SES and MED services. Pre-pandemic, family income positively correlated with access to SES and ABA services (i.e., higher income families accessed more SES and ABA services) and negatively correlated with MH and MED services (i.e., lower income families accessed more MH and MED services). Interestingly, one year after the pandemic, positive correlations were seen between family income and receipt of multiple services including SES, SLT, and ABA services (i.e., higher income families accessed more of these services) and negatively correlated with MH services (i.e., lower income families accessed more MH services). Pre-pandemic, minority status negatively correlated with access to SLT services (i.e., non-white families accessed more SLT) but positively correlated with access to MH services (i.e., white families accessed more MH services). Post-pandemic, SLT and ABA trends were similar to pre-pandemic trends (i.e., non-white families accessed more SLT and ABA). While there were no differences pre-pandemic, one year after the pandemic, ethnicity was positively associated with SLT receipt (i.e., Hispanic families received more SLT services than non-Hispanic families). Geographic location was positively correlated with access to ABA pre-pandemic and access to SLT post-pandemic (i.e., living in urban area was associated with higher ABA and SLT receipt). Sex did not correlate with access to any service at either timepoints.

**Table 5:** Spearman correlations between demographic variables (rows 2–7) and parent-reported impact of COVID-19 on services received (columns 3–8) for pre-pandemic data (first entry in each data cell) and data from march 2021 (12 months after pandemic onset—second entry in each data cell). Correlations with *p* values <0.0014 after Bonferroni corrections are highlighted in yellow; and correlations with *p*-values >0.0014 and <0.01 are highlighted in green.

Services received/factors	Time point	SES	SLT	PT/OT	ABA	MH	MED
Age group	T2	0.157	−0.272	−0.297	−0.2	0.313	0.13
	T5	NS	−0.278	−0.304	−0.224	0.246	NS
Income level	T2	0.054	NS	NS	0.094	−0.082	−0.128
	T5	0.087	0.085	NS	0.067	−0.065	NS
Minority status	T2	NS	−0.064	NS	NS	0.075	NS
	T5	NS	−0.105	NS	−0.095	NS	NS
Ethnicity	T2	NS	NS	NS	NS	NS	NS
	T5	NS	0.066	NS	NS	NS	NS
Geographic location	T2	NS	NS	NS	0.069	NS	NS
	T5	NS	0.069	NS	NS	NS	NS
Sex	T2	NS	NS	NS	NS	NS	NS
	T5	NS	NS	NS	NS	NS	NS

NS, not significant; SE, special education; PT/OT, physical and occupational therapy; SLT, speech and language therapy; ABA, applied behavior analysis; MH, mental health; MED, medical.

## 4 Discussion

### 4.1 Overall results

This analysis examined the recovery in services received by children with ASD using an online parent survey completed by the SPARK cohort in the first year of the COVID-19 pandemic. The data extracted from the SPARK cohort is a fairly well-represented sample of children with ASD across the United States. Overall, SES had fully recovered one year after the pandemic onset, and MH and MED services had superseded the baseline levels, while SLT, PT/OT and ABA services had not fully recovered. Specifically, ABA services had recovered for some but not all subgroups. Among demographic factors, age, income, minority status, ethnicity, and geographic location were associated with service access pre/post-pandemic. Pre-pandemic, age was associated with all services, with younger children receiving more SLT, PT/OT, and ABA and older children receiving more SES, MH, and MED services; however, post-pandemic, these trends continued for SLT, PT/OT, ABA, and MH only. In terms of service recovery, service access to SLT, PT/OT, and ABA recovered more in younger children compared to older children. In general, families with higher income received more SES, SLT, and ABA and lower income families accessed more MH and MED services. One year after the pandemic, most income groups recovered in their access to SES, MH, and MED services but access to SLT, PT/OT, and ABA services did not recover. Nevertheless, SES, SLT, and ABA services continued to be greater for higher income families compared to lower income families whereas lower income families accessed more MH services. While white and non-white families showed service recovery for MH and MED services; neither showed recovery in access to PT/OT services. SES recovered more for white families whereas SLT and ABA services recovered more for non-white families. While there were no pre-pandemic differences, Hispanic families received more SLT and had greater recovery compared to non-Hispanic families, post-pandemic. Compared to rural families, urban families received more ABA in general and had ABA service recovery after one year but this was not seen in the rural families. Lastly, one year following the pandemic, PT/OT services had not fully recovered in any of the subgroups based on age, income levels, race/ethnicity, location, or sex. The overall findings indicated that health inequities across various subgroups either remained the same, exacerbated or reversed during the pandemic.

### 4.2 Recovery across service types

In terms of school-based services, before the pandemic, access to SES and SLT was greater than PT/OT, and then ABA services. One year after the pandemic, in March 2021, SES and ABA seemed to have recovered but SLT and PT/OT services had not reached pre-pandemic levels. Specifically, PT/OT services significantly lagged in recovery for the majority of subgroups. ABA services only lagged in recovery for about half of the subgroups. Pre-pandemic, compared to school-based services such as SES, SLT, PT/OT fewer children with ASD received MH and MED services. However, one-year post-pandemic, MH and MED services superseded their pre-pandemic levels. The rise in access to MH and MED services may be due to the worsening in children's ASD severity following the sudden loss in therapeutic services after prolonged lockdowns and social distancing policies. Another possible explanation is that MED and MH services were mainly provided through community clinics (outside of school settings) and hence, were less susceptible to school closures. After lockdowns subsided, community-based clinics may have recovered sooner than schools due to the small-scale nature of clinics (i.e., involved fewer clinicians or provided one-on-one services). It may also be that MH and MED services, are more prescription/conversation-based and require less hands-on interactions, and hence, are more amenable to shifting to telehealth formats. It is also possible that telehealth-based models of MED and MH services were established long before the COVID-19 pandemic for greater access to rural areas and were easily utilized during the pandemic due to preexisting infrastructure and functional insurance reimbursement policies (38–42). Similar to MED and MH services, telehealth-based, parent-mediated delivery ABA models were already being tested and developed before the pandemic and hence, the transition to telehealth ABA services was also accelerated by the COVID-19 pandemic (43–45). Hence, apart from the recovery in school-based ABA services, alternative formats may have restored ABA services in certain subgroups. Note that for certain subgroups (e.g., older children, rural families, white families, older children and middle-income groups) ABA services were still lagging in recovery one year after the pandemic.

The most prominent finding across all subgroups was the significant lag in service recovery in PT/OT services. Conventional PT/OT services, often utilize materials such as larger play equipment and spaces such as the school gym or clinic facilities. It also requires more hand-on-hand assistance from clinicians making it more challenging for PT/OT to transition to telehealth formats. In addition, PT/OT interventions are often provided to children with ASD needing significant support and screen-based therapies requiring attentional focus can be challenging for younger children as well as those needing more support. It is possible that these barriers reduced access to PT/OT services even one-year post-pandemic. However, recent clinical reports confirm that telehealth can be implemented by developing creative play activities using home-based supplies, by delivering supplies and training parents prior to session delivery (45, 46). Telehealth OT via parent-mediated coaching was also found to be highly acceptable for parents with children of ASD (47). However, there is a need for more research in the area of telehealth PT/OT services to identify effective ways to provide services to children with developmental disabilities, in general.

Before the pandemic, there was limited evidence for how SLT could be delivered through parent-implemented remote coaching in children with ASD. Sutherland et al. conducted a systematic review of 14 studies examining telehealth delivery of mental health counseling and ASD diagnosis/assessment efficacy and found their efficacy to be similar to that of face-to-face interaction models; however, they only reported one SLT intervention using a single case report (48). Similar to PT/OT, before the pandemic, children with ASD mainly received SLT services during their regular school day (7, 8, 49). While school-based services switched to remote formats, telehealth SLT was perhaps not easily adopted for multiple reasons. SLT heavily relies on the use of language and sound during treatment which made it more susceptible to technological issues such as poor audio-visual quality, internet connectivity and background noise, resulting in more implementation barriers. In addition, telehealth formats of SLT and PT/OT services require greater parental involvement for them to be meaningfully delivered to children needing more support who have attention difficulties. For all these reasons, transitioning SLT and PT/OT services to alternative formats may have been more challenging.

### 4.3 Differences in service recovery based on age

Younger children received more SLT, PT/OT, and ABA but less SES, MH, and MED services compared to older children. This fits with the literature reporting that younger children receive more therapy services (SLT, PT/OT, and ABA) which decline with age and instead older children/adolescents are more likely to receive MH and MED services compared to younger children (6, 7, 12). Hence, it is not surprising that service access of younger children was restored for the services that they most received pre-pandemic (i.e., SLT, PT/OT, and ABA). Early interventions through SLT, PT/OT, ABA are mandated by the IDEA law and therefore, younger children accessed more SLT, PT/OT and ABA than older children to meet their urgent developmental needs. It is also reported that older children/adolescents with ASD do not receive enough SLT and PT/OT (i.e., important for communication and functional skill development) resulting in significant unmet needs in many young adolescents and adults (6, 50, 51). In short, our findings indicated that older children/adolescents with ASD faced even greater service losses post-pandemic, as they were already facing unmet service needs related to SLT and PT/OT services before the pandemic.

In contrast, we also found increased use of MH and MED services in older children before and after the pandemic. This is consistent with the previous findings that older children (ages 15–17 years) use more individual counseling and group therapy than younger children (12). In addition, older youth with high-functioning ASD had greater MH and MED service needs such as mental health counseling, psychotherapy, and prescription medication use due co-occurring conditions that may develop with age, e.g., anxiety, attention deficit, depression, and other psychiatric conditions (5, 52, 53). Additionally, the social distancing requirements and drastic changes to daily routines/school schedules may have worsened children's ASD severity and in turn increased their need for MH and MED services.

### 4.4 Differences in service recovery based on income

Past studies have reported that lower socioeconomic status is associated with a decreased likelihood of receiving special education services and ABA services (15–17) and that families with higher SES were more likely to enroll/advocate for their children to receive PT/OT and ABA services within or outside of school (21). Although our result of higher income families receiving fewer MH services compared to lower income families seems non-intuitive at the outset, it is possible that higher income families were less negatively impacted during the pandemic and did not seek out as many MH services. The finding of MED service receipt increasing across all income groups, was different from past studies. For example, Liptak et al. (18) and Lokhandwala et al. (19) found that families from lower SES reported reduced access to primary healthcare and ASD treatment services as well as lower rates of hospitalization/underutilization of services. It is possible that in general children with ASD were not receiving the required SES, PT/OT, and ABA as part of their regular school day and that may have worsened their child's ASD symptoms and led to them to seeking more MED services.

### 4.5 Differences in service recovery based on race/ethnicity

One year after the pandemic, SLT services had not fully recovered in white families, whereas SES had not fully recovered for non-white families. Hispanic families received more SLT compared to non-Hispanic families. Lastly, PT/OT services did not recover for any of the subgroups based on race/ethnicity. Previous studies have reported that children with ASD from white families are known to receive critical MH services much earlier (54). However, our findings suggested white families received less SES, SLT, and ABA seems counterintuitive. We speculate that white families had more access and resources through homeschooling and were unable to access as many “related services” such as SLT and ABA that they previously received from public schools. In addition, post-pandemic concerns about getting COVID-19 may have prevented white families from accessing community or school-based services even as late as March 2021 as pediatric vaccines were unavailable at the time.

### 4.6 Differences in service recovery based on geographic location

We found that urban families received more SLT and ABA; whereas rural families received more MH services pre-pandemic. Post-pandemic service recovery was seen in majority of services for both urban and rural families; except SLT, PT/OT, and ABA services. ABA only fully recovered in urban families and PT/OT as well as SLT services did not recover for either subgroups. This fits with what we know from previous studies that children living in metropolitan (vs. rural areas) have greater access to SLT and ABA/behavioral therapies at schools and in the community (7, 16). This may have made it easier for urban family to recover, one-year post-pandemic. Rural families mainly access services through their public schools; which were either closed or not providing adequate services due to staff/teacher shortages and limited resources in adapting to telehealth interventions. Together, this may explain the slower service recovery in rural families, one-year post-pandemic.

### 4.7 Limitations, conclusions, and future directions

Through this analysis, we offer a historical record, of how children with ASD faced service loss and partial service recovery in the first year after the COVID-19 pandemic. Multiple demographic factors were associated with services received. Although this was a relatively larger, representative sample of children with ASD in the US, the proportion of certain minorities was lower—non-white (10%) and Hispanic (15%). In addition, this was a survey study requiring families to respond at multiple timepoints which in turn led to substantial missing data over time. While these results provide a historical record of service disruptions and recovery in the first year of the pandemic, they should be interpreted with caution for subgroups with smaller samples. Other child and parent-related factors could also affect service recovery following the pandemic and will be reported elsewhere. Lastly, the SPARK COVID-19 impact survey ended after the first year of the pandemic; hence, there is no record of how services evolved and bounced back to normalcy in the school year that followed; when COVID-19 vaccines became more available to the public.

In the first year of the pandemic, school and medical systems made substantial efforts to fully restore all therapeutic services and not just special education, mental health, and medical services; which mostly recovered; 1-year post-pandemic. However, PT/OT services showed the most lag in service recovery followed by SLT, and lastly ABA. In the future, more research is needed to study the feasibility, acceptability, and health outcomes using alternative formats of PT/OT and SLT services (telehealth-based, community-based, or home-based) as well as parent or aide-mediated interventions (as opposed only relying on in-person, school-based services). We also recommend families and insurance companies to continue accessing community-based/home-based services that can be covered through health insurance to fill intervention gaps that exist within school systems. Policymakers should enforce laws that support alternative therapeutic formats outside of school systems, including community clinics, home-based services, as well as telehealth services for children with ASD and other disabilities. The COVID-19 pandemic tested the strength of our health service systems, revealed the vulnerabilities of therapeutic services offered to children with ASD and other disabilities in the US, and indicate the urgent need for greater diversity in therapeutic service formats.

## Data Availability

Publicly available datasets were analyzed in this study. This data can be found here: https://base.sfari.org.
